# Anthocyanin in the Vacuole of Red Onion Epidermal Cells Quenches Other Fluorescent Molecules

**DOI:** 10.3390/plants8120596

**Published:** 2019-12-12

**Authors:** David A. Collings

**Affiliations:** 1 School of Environmental and Life Sciences, The University of Newcastle, Callaghan, NSW 2308, Australia; david.collings@newcastle.edu.au; 2 School of Biological Sciences, The University of Canterbury, Private Bag 4800, Christchurch 8140, New Zealand

**Keywords:** *Allium cepa*, anthocyanin, fluorescence quenching, onion, onion epidermis

## Abstract

Peels from the inner epidermis of onion bulbs are a model system in plant cell biology. While the inner epidermis of red onions is characteristically white, small patches of cells sometimes redden, containing vacuolar anthocyanin. This study investigated the spectroscopic properties of these anthocyanic cells. When fluorescent dyes were loaded into the vacuole of onion epidermal cells, the anthocyanic cells showed decreased dye fluorescence. This decrease was observed for fluorescein and carboxyfluorescein that are pumped into the vacuole by anion transporters, for acridine orange which acid loads into the vacuole, and for the fluorescent sugar analogue esculin loaded into the vacuole by sucrose transporters. Similar decreases in carboxyfluorescein fluorescence were observed when dye was loaded into the vacuoles of several other plant species, but decreases were not observed for dyes resident in the tonoplast membrane. As cellular physiology was unaffected in the anthocyanic cells, with cytoplasmic streaming, vacuolar and cytoplasmic pH not being altered, the decreased dye fluorescence from the anthocyanic cells can be attributed to fluorescence quenching. Furthermore, because quenching decreased with increasing temperature. It was concluded, therefore, that vacuolar anthocyanin can statically quench other fluorescent molecules in vivo, an effect previously demonstrated for anthocyanin in vitro.

## 1. Introduction

Anthocyanins are flavonoid pigments present in the vacuoles of higher plant cells that generate a wide diversity of colours ranging from orange and red to violet and blue. Anthocyanins are composed of an anthocyanidin core that consists of two phenyl rings that may be variously substituted. Although about 20 different core structures are known, the main six cores are cyanidin, delphinidin, malvidin, pelargonidin, peonidin and petunidin, with these named for the plants from which they were first isolated. The anthocyanidin core is modified by the addition of one or more sugar residues, typically at the C3, C5 and/or C7 position, and these sugars can be further modified. The naming of the more than 500 distinct anthocyanins that have been identified reflects both components with, for example, cyanidin-3-glucoside being composed of a cyanidin core linked to glucose [1]. Variations within the anthocyanidin core and the attached sugars explain some of the different spectral properties of anthocyanins. Increased hydroxylation present in the anthocyanidin core (for example, delphinidin) generates pigments with colours shifted towards blue whereas glycosylation results in reddening. However, the colours formed by anthocyanins will also depend on both the vacuolar environment, and on the secondary and tertiary structures formed by the anthocyanins. In the acidic environment of the plant vacuole, anthocyanins exist in an equilibrium of six states. These include coloured flavylium cation and quinoidal species, and various other non-coloured species. The stability of these forms is pH-dependant, which means that anthocyanin colour, and the colour of the plant, will depend upon vacuolar pH. Anthocyanin colour may also vary because anthocyanins can interact with themselves, stacking to form super-molecular structures, and can interact with other flavonoid pigments, stabilising the coloured forms at acidic pHs, and also interact with metal ions [1,2,3,4].

Like many flavonoids, anthocyanins can be weakly autofluorescent with in vitro excitation and emission peaks in the UV [4,5]. However, in vivo excitation of anthocyanins in both *Arabidopsis thaliana* and onion (*Allium cepa*) results in red fluorescence, and has allowed for direct visualisations of vacuole structure and dynamics [6,7,8].

The flavonoid biosynthetic pathway, a complex series of reactions in which coumaroyl-CoA is sequentially modified to form various end-products, results in the formation of anthocyanins [9]. The enzymes controlling this pathway vary slightly between species resulting in the variability in the anthocyanidin core and attached sugars. Following their synthesis in the cytoplasm, the vacuolar transport of anthocyanins occurs through several pathways. These include ER-derived vesicles [6,10] and a tonoplast-bound glutathione *S*-transferase-like transporter that sequesters various glutathione conjugates and anthocyanins into the vacuole [11,12]. The mechanism(s) underlying this second process are unclear as anthocyanin-glutathione conjugates remain undiscovered [12]. The multi-drug resistance-like protein is another membrane-bound transporter known to transport anthocyanin into maize vacuoles [1].

It is thought that anthocyanins play multiple roles in plants. Anthocyanins provide a visual cue for the animals that pollinate and disseminate flowers and fruits. Furthermore, because they can be induced by various stresses, anthocyanins thought to be bio-protectant molecules [9,13] acting directly against reactive oxygen species (ROS) that are formed by the plant during photosynthesis and respiration [14]. The anthocyanin flavylium cation effectively reduces the formation of free radicals, and scavenges those oxygen radicals that do form, both in vitro [15,16] and in vivo [17]. For anthocyanins to act as antioxidants, they need to interact with ROS. The separation of anthocyanins in the vacuole from the production of most ROS, produced by the mitochondria and chloroplasts [18], has led to debate concerning the physiological significance of anthocyanins as antioxidants [17,18]. Thus, while anthocyanins act as anti-oxidants in vivo, because anthocyanins in the vacuole are spatially separated from the generation of ROS in the cytoplasm and organelles, it remains uncertain as to whether they do function as antioxidants in vivo. Horopito (*Pseudowintera colorata*), a New Zealand native species that shows natural variegation in anthocyanin in its leaves has been used to demonstrate that anthocyanin, even though located in the vacuole, can act as an anti-oxidant in vivo. Fluorescence imaging using cytoplasmically-localised dichlorofluorescein as a hydrogen peroxide reporter showed that wound-induced H_2_O_2_ was dissipated faster in tissues that contained red, anthocyanic epidermal and mesophyll cells than in regions of leaf that were green [19]. There are, however, some limitations in this study as it was conducted in whole leaves which had been sectioned, creating wounding, to give access to reagents.

Red onions contain anthocyanins in their epidermal cells, with the primary component of the 25 anthocyanins produced being cyanidin-3-*O*-glucoside, although a complex mixture of other cyanidin and peonidin derivatives are also present [20,21]. In this paper, this complex mixture of different anthocyanin species is referred to generically as “anthocyanin.” This complex mixture of anthocyanins is autofluorescent, with onion epidermal vacuoles showing a broad range of excitation wavelengths, and having an emission spectrum showing a single, red emission peak at around 630 nm [7,8]. The contribution of the different anthocyanins present in onion to the emission spectrum remains unknown. As the inner epidermis of the leaves of onion bulbs can be readily removed as an epidermal peel, with these cells remaining alive for extended periods of time without wound responses, onion epidermal cells are an attractive system for studying anthocyanin. This inner epidermis in normally white, although anthocyanins can be induced by environmental and genetic means [8]. On occasions, however, cells at either ends of the bulb can show anthocyanin in a highly variable patterns. Thus, anthocyanic red cells are interspersed between anthocyanin-free white cells.

The initial goal of this study was to load dye into the vacuole of red onion cells to test whether anthocyanin can act as an anti-oxidant in vivo. As anti-oxidants such as *p*-phenylenediamine are often added to samples viewed by fluorescence microscopy where they act to prevent photobleaching [22], it was thought that dyes loaded into the vacuole might bleach less rapidly when compared to adjacent cells lacking anthocyanin, thus demonstrating anthocyanin’s antioxidant abilities. However, this study showed this initial model to be simplistic. Multiple dyes loaded into the vacuole showed marked reductions in fluorescence in anthocyanin-containing cells. As the physiology of these cells was similar to anthocyanin-free cells, it can be concluded that anthocyanin directly quenches other fluorescent molecules. Furthermore, because this quenching is reduced at higher temperatures, the quenching is therefore static rather than dynamic.

## 2. Results

### 2.1. Measuring Anthocyanin in Onion Epidermal Cells

**Figure 1:**
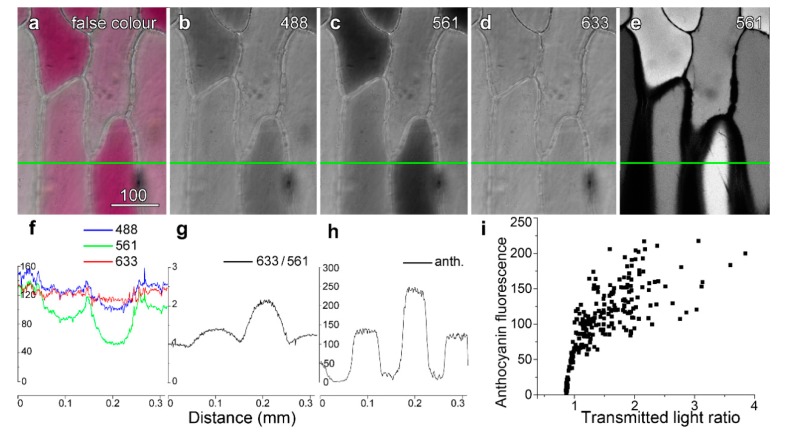
Quantifying vacuolar anthocyanin. Images were recorded sequentially in line-by-line mode using four-fold averaging. A false colour transmitted light image (**a**) was created using (**b**) blue (488 nm), (**c**) green (561 nm) and (**d**) red (633 nm) light images, with these collected at the same time as high-intensity 561 nm green excitation to excite anthocyanin (**e**). Intensity profiles were generated along the green line with ImageJ, and plotted for the three different transmitted light wavelengths. The intensity plot for the green image dropped markedly in the red cell, consistent with green light being absorbed, whereas intensity in the red image dropped only slightly. (**f**) Dividing the red intensity by the green intensity (T633/T561) provided a convenient measure of the anthocyanin present in the cell, in which variations present in both images such as cell walls and organelles were reduced. (**g**) Anthocyanin fluorescence intensity. (**i**) Anthocyanin showed a strong but non-linear correlation with the transmitted light ratio (T633/T561). Bar in (**a**) = 100 µm for (**a**–**e**).

The inner epidermis of red onion cells, adjacent to the ends of the bulb, often contains occasional patches of red, anthocyanic cells interspersed between paler and non-coloured cells. Cells that were more strongly red in colour (Figure 1a), absorbing green and blue but not red light (Figure 1b–d), fluoresced more strongly from the vacuole (Figure 1e). Two methods were developed to quantify the degree of pigmentation. These were measurements of the ratio of the transmitted light images collected with non-absorbing red light (633 nm) divided by the more strongly absorbed green light (561 nm) (Figure 1f,g), and direct measurements of anthocyanin fluorescence intensity collected under specific imaging conditions (Figure 1h). Because there was some variability in quantification between experiments conducted on different days, due in part to variations in laser output and rates of dye loading, the data presented in this and subsequent Figures are limited to representative experiments conducted on multiple epidermal peels conducted in a single day.

When quantified, anthocyanin fluorescence correlated well to the transmitted light ratio, although the non-linear relationship suggests that at higher anthocyanin concentrations, self-shading and absorbance effects may have reduced the anthocyanin fluorescence (Figure 1i). This correlation between the fluorescence and absorbance ratios for anthocyanin held even though the measurements related to different geometries. Anthocyanin fluorescence was measured in a single focal plane whereas the transmitted light ratio measured anthocyanin through the depth of the cell.

### 2.2. Anthocyanic Cells Show Reduced Fluorescence Vacuolar Dyes

**Figure 2:**
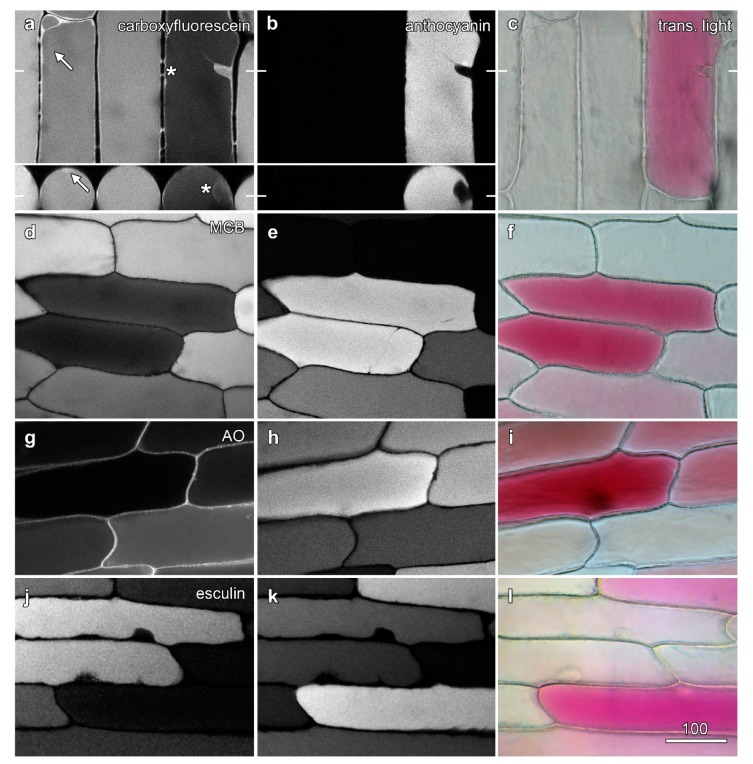
Fluorescence from dyes in the vacuole is lower in the presence of vacuolar anthocyanin. Onion epidermal peels were loaded with dyes (6 h) and imaged by confocal microscopy, with fluorescence images of the dyes (left column; **a**,**d**,**g**,**j**) collected concurrently with anthocyanin fluorescence (central column; **b**,**e**,**h**,**k**) and colour transmitted light images (right column; **c**,**f**,**i**,**l**). (**a**–**c**) Carboxyfluorescein imaged with blue excitation (488 nm), with cytoplasmic fluorescence highlighted in a red cell (asterisk) arrow). The inset shows a single vertical section (XZ) imaging generated at the location marked by bars in (**a**). (**d**–**f**) Monochlorobimane (MCB) imaged with violet excitation (405 nm). (**g**–**i**) Acridine orange (AO) imaged with blue excitation (488 nm). (**j**–**l**) Esculin imaged with violet excitation (405 nm). Bar in (**i**) = 100 µm for all images.

Carboxyfluorescein diacetate, a non-fluorescent membrane-permeable molecule, can diffuse into the cytoplasm where esterases convert it into a membrane-impermeable fluorescent molecule carboxyfluorescein which is then slowly pumped by anion transporters into the vacuole [23,24]. Unlike unpigmented epidermal cells where carboxyfluorescein labelling was generally even, variegated onion epidermal cells exhibited large differences in the amount of vacuolar carboxyfluorescein fluorescence generated by blue (488 nm) excitation. The fluorescence was weaker in cells that contained anthocyanin, as shown by both colour transmitted light and red anthocyanin fluorescence generated with green (561 nm) excitation (Figure 2a). This decrease was not due to lower loading of carboxyfluorescein into the anthocyanic cells, as cytoplasmic fluorescence was similar whether vacuolar anthocyanin was present or not (Figure 2a, arrow). Moreover, this decrease in carboxyfluorescein fluorescence was not due to anthocyanin re-absorbing and filtering carboxyfluorescein fluorescence. Samples re-imaged in a vertical (XZ) plane through the epidermis showed minimal carboxyfluorescein fluorescence throughout the vacuole of anthocyanic cells including at the upper surface where reabsorption by anthocyanin should be less problematic (Figure 2a, inset). Similar differences in loading were also observed in several other esterase-released including fluorescein diacetate, BCECF-AM ester and Cell Tracker green (5-chloromethylfluorescein diacetate) (Figure S1a–c).

Several further dyes that load into the vacuole were also tested. Monochlorobimane is a probe used to detect glutathione, as its reacts with thiols to produce a fluorescent product that is also pumped in to the vacuole [25,26]. Cells treated with monochlorobimane, in which the vacuole fluoresced blue following violet (405 nm) excitation, contained less anthocyanin (Figure 2d). Similarly, Cell Tracker blue is a chloromethylcoumarin derivative that reacts with thiols to produce a fluorescently-tagged molecule that is then pumped into the vacuole [27]. It too fluoresced less strongly in cells containing anthocyanin (Figure S1d).

The esterase-activated dyes, and the products of monochlorobimane and Cell Tracker Blue, all load via the same or similar transport systems. This has been demonstrated with the drug probenecid, an inhibitor of anion transporters, that blocks the vacuolar accumulation of carboxyfluorescein [23], monochlorobimane [26] and ZFR-CMAC, a molecule cleaved by proteases to generate Cell Tracker Blue [26]. Although it would seem unlikely, this transporter system might vary between non-coloured and coloured cells, or become saturated through the loading of anthocyanins/Therefore, fluorescent dyes were tested that load by a different mechanism. Acridine orange loads into the vacuole as a weak acid [24], and also showed a similar reduction of fluorescence in anthocyanic cells (Figure 2g). Similarly, esculin, a fluorescent sucrose analogue that is a coumarin glucoside and that is pumped across the plasma membrane and then into the vacuole by sucrose transporters [28] also loaded into the vacuole of onion epidermal cells where it too showed less fluorescence in anthocyanic cells (Figure 2j).

### 2.3. Anthocyanin Reduces Carboxyfluorescein Fluorescence in the Vacuole of Other Species

The inverse relationship between vacuolar carboxyfluorescein fluorescence and anthocyanin fluorescence was also present in other plants. Partial epidermal peels of variegated leaves from *Coleus*, *Neoregelia* and *Dracaena* (Figure S2a–c) and the patterned flower petals of *Cyclamen* and *Aquilegia* (Figure S2d,e). However, while the effect could be observed in these plants, the increased difficulty in preparing and labelling these tissues precluded quantitative studies.

### 2.4. Anthocyanic Cells Show Even Labelling of Tonoplast-Specific Dyes

**Figure 3:**
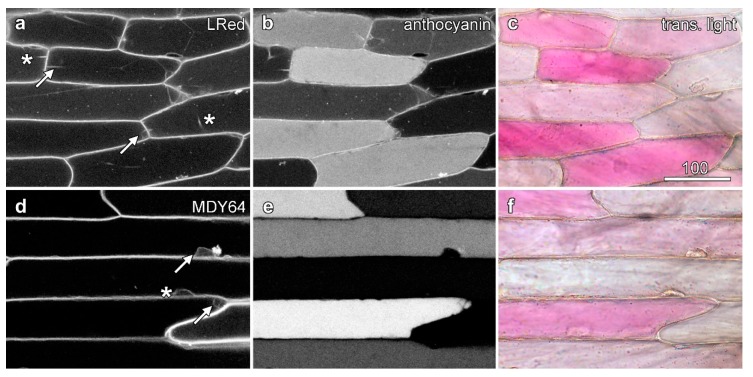
Tonoplast labelling is unaffected by the presence of anthocyanin. Epidermal peels loaded with dyes were imaged with sequential scanning. Images are confocal optical sections of dye fluorescence (left column; **a**,**d**), with the tonoplast labelling in anthocyanic cells indicated with arrows and in white cells with asterisks, anthocyanin fluorescence (central column; **b**,**e**), and a colour transmitted light image (right column; **c**,**f**). (**a**–**c**) Lysotracker Red DND-99 (LRed) imaged with green excitation collecting shorter wavelength red fluorescence, with the longer wavelength red fluorescence collected from anthocyanin with some bleed-through of the Lysotracker. (**d**–**f**) MDY-64 imaged with blue (473 nm) excitation. Bar in (**a**) = 100 µm for all images.

Two dyes that target to the onion tonoplast membrane, MDY-64 and Lysotracker Red DND-99 [29], loaded rapidly into epidermal cells following short loading times of between 5 and 10 min. Lysotracker Red acid which acid loads in a similar way to acridine orange [27] did not show reduced fluorescence in anthocyanic cells (Figure 3a). Similarly, the tonoplast marker MDY-64 also failed to show reduced labelling in anthocyanic cells (Figure 3d).

### 2.5. Physiological Variations Do Not Account for Variations in Epidermal Cell Fluorescence

Dyes that load into the vacuole through different mechanisms all show a reduction in fluorescence in anthocyanin-containing cells. It is possible that changes in cellular physiology and biochemistry are present in cells containing anthocyanin which limited dye uptake. This was investigated in several ways.

**Figure 4:**
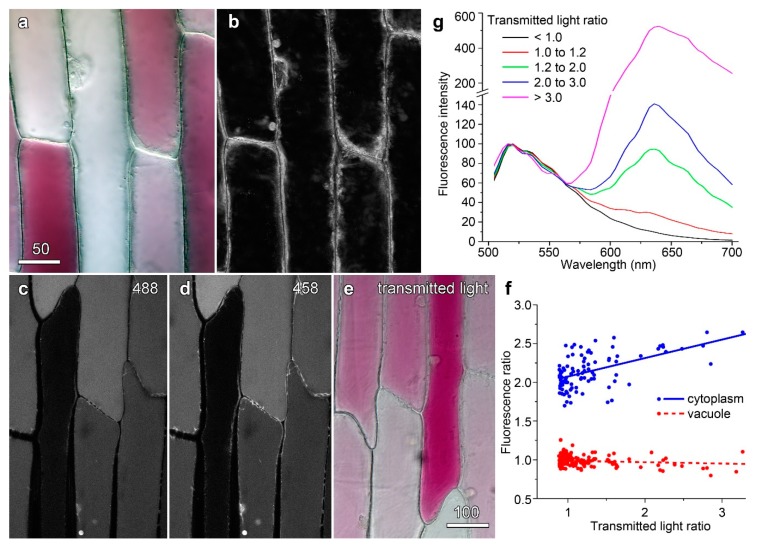
Anthocyanic cells show no differences in cell physiology compared to less strongly coloured neighbouring cells. (**a**,**b**) Analysis of cytoplasmic streaming. (**a**) Colour transmitted light imaging. (**b**) An average image was determined for the entire time-lapse sequence (40 images over 2 min), and each image divided by the average image. The image shown is a maximum projection of this processed time-course, which highlights areas of dynamism. No differences were observed in the dynamics of anthocyanin-containing cells. (**c**–**f**) Analysis of intracellular pH using carboxyfluorescein ratio imaging. (**c**) 488 and 458 nm (**d**) excitation of CFDA with (**e**), anthocyanin imaging using colour transmitted light. (**f**) Correlations of vacuolar and cytoplasmic fluorescence ratios (488 nm image divided by 458 nm) with anthocyanin (transmitted light ratio). (**g**) Fluorescence emission for carboxyfluorescein was unmodified in cells with different anthocyanin contents. Cells were pooled into five groups based on separate measurements of the transmitted light (T633/T561) ratio and normalised curves calculated for N ≥ 4 cells. The emission peak at 520 nm corresponds to carboxyfluorescein while the peak at 635 is anthocyanin. Bar in (**a**) = 50 µm for (**a**,**b**); bar in (**e**) = 100 µm for (**c**–**e**).

Cytoplasmic streaming is often taken as an indicator of plant cell health [30]. Following colour transmitted light imaging to establish variations in anthocyanin (Figure 4a), cytoplasmic streaming was observed using red light. An image showing the difference between each image and an average projection (Figure 4b) gave no indication that anthocyanic cells were any less active than their less red neighbours. Vacuolar and cytoplasmic pH have also been investigated to determine whether redder cells have altered pH homeostasis. Anthocyanins are natural pH indicators, changing from red at acidic pHs through blue to green at less acidic and neutral pHs, with these colour changes known to reflect vacuolar pH [31]. It has previously been shown that onion epidermal cells showing a wide range of anthocyanin levels have constant ratios of absorbance for red, green, and blue light [30]. This consistency in ratio indicates the cells are the same colour and, thus, that the vacuoles have the same pH irrespective of anthocyanin level. Moreover, changing vacuolar pH with the weak base methylamine [32,33], making the vacuole less acidic, caused change to absorbance ratios [30].

Cellular pH can also be directly probed with carboxyfluorescein as it is a dual excitation ratiometric pH indicator. Its fluorescence is strongly pH-dependent when excited at 488 nm, fluorescing more strongly in neutral conditions than acidic environments, but nearly pH-independent when excited at 458 nm close the isobestic point at around 460 nm [27]. When carboxyfluorescein was excited at 488 nm, cortical cytoplasm was visible around the edges of cells (Figure 4c) but the distinction between vacuole and cytoplasm was not clear when excited at 458 nm (Figure 4d).No differences in pattern were seen between cells that were strongly red in colour, and those that were less strongly coloured (Figure 4e).When the images were ratioed (fluorescence caused by 488 nm excitation divided by fluorescence caused by 458 nm excitation), differences in pH were seen between the cytoplasm (a ratio of about two, near neutral) and vacuole (a ratio of about one, more acidic). Quantification of the ratio effect for approximately 100 cells showed that cytoplasmic pH may have increased slightly in the redder cells but that there was no change in ratio for the vacuole consistent with the vacuolar pH being constant (Figure 4f). Therefore, it would seem more likely that reductions in fluorescence from dyes such as fluorescein and carboxyfluorescein occur at a biophysical level, and that anthocyanin directly modulated the fluorescence of the dyes. Consistent with this, fluorescence emission spectra from carboxyfluorescein were similar in anthocyanin-free and anthocyanin-containing cells, indicating that the physical surrounds of the dye were not greatly changed (Figure 4g). These experiments all suggest that the physiology of the red and white cells is similar and, therefore, that the reductions in dye fluorescence are due to quenching by anthocyanin.

### 2.6. Quantifying Anthocyanin-Induced Quenching in the Vacuole

**Figure 5:**
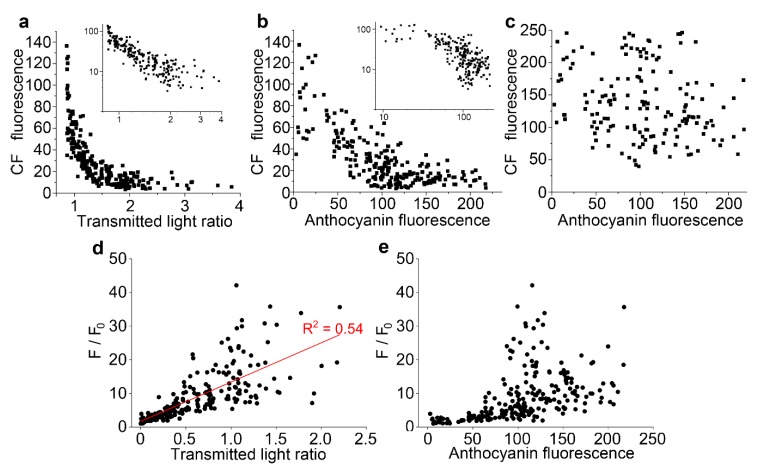
Vacuolar fluorescence of carboxyfluorescein is lower in the presence of anthocyanin. For individual cells from multiple epidermal peels in a single representative experiment, anthocyanin and carboxyfluorescein fluorescence were measured in arbitrary units, with anthocyanin also measured as a transmitted light ratio, the image intensity at 633 nm divided by the intensity at 561 nm. (**a**) Vacuolar carboxyfluorescein showed a strong negative correlation with the transmitted light ratio. The inset shows the same dataset replotted on a double-log plot. (**b**) Vacuolar carboxyfluorescein showed a negative correlation to anthocyanin fluorescence. The inset shows the same dataset replotted on a double-log plot. (**c**) Cytoplasmic carboxyfluorescein did not correlate to anthocyanin fluorescence. (**d**,**e**) Stern–Volmer plots, used to investigate the quenching of fluorescence, correlate maximum dye fluorescence divided by the dye fluorescence (F/F_0_) against anthocyanin concentration: when plotted against transmitted light ration (**d**), a straight line was observed whereas plotting against anthocyanin fluorescence did not give a straight line (**e**).

The link between anthocyanin and the quenching of fluorescent dyes in the vacuole was quantified using carboxyfluorescein diacetate. In cells loaded with carboxyfluorescein diacetate, there was a strong negative correlation between vacuolar green fluorescence from carboxyfluorescein and both the transmitted light ratio (Figure 5a) and anthocyanin fluorescence (Figure 5b). Double log plots of these data showed a near straight-line response for the relationship between carboxyfluorescein and the transmitted light ratio (Figure 5a, inset). There was, however, no link between carboxyfluorescein fluorescence in the cytoplasm of cells and the anthocyanin fluorescence from the vacuole (Figure 5c). Further analyses quantified similar links between vacuolar fluorescence of dyes and anthocyanin in cells labelled with both monochlorobimane (Figure S3a) and fluorescein (Figure S3b).

The correlations between carboxyfluorescein fluorescence and anthocyanin was investigated with Stern–Volmer plots, often used to characterise fluorescence quenching in vitro [34], and in which the fluorescence from a fluorescent species in the absence of quencher, divided by the molecule’s fluorescence in the presence of the quencher (F/F_0_), generate a straight line when plotted against the concentration of the quencher. For these analyses, carboxyfluorescein data were replotted against anthocyanin concentration, taken either as the raw anthocyanin fluorescence (Figure 5e) or a modification of the transmitted light ratio, defined as the ratio minus the minimum transmitted light ratio recorded which was assumed to be equivalent to no anthocyanin (Figure 5d). The expected straight line response was generated, consistent with molecular quenching, when the plots were conducted against anthocyanin measured with the transmitted light ratio (R^2^ = 0.54) (Figure 5d). However, a non-linear response was present when data were plotted against anthocyanin fluorescence (Figure 5e). This non-linear response would be consistent with reduced fluorescence at high anthocyanin concentrations because of self-shading and absorbance effects, as indicated by the non-linear relationship between the two anthocyanin measurements (Figure 1i). Subsequent analyses were only conducted against the transmitted light ratio.

Stern–Volmer plots were also applied to monochlorobimane and fluorescein quenching by anthocyanin, and again giving straight line relationships (R^2^ = 0.87 and 0.53 respectively) (Figure S3b,d).

### 2.7. The Effect of Temperature on Quenching

Molecular quenching can occur in two different ways. These mechanisms are referred to as static quenching, in which direct and continued interactions between the quencher and the fluorescent molecular prevent excitation from occurring, and dynamic quenching where the excited fluorophore loses energy to the quencher through an energy transfer process. One way in which static and dynamic quenching can be differentiated is through their temperature dependence. Because static quenching relies on the formation of a ground state complex between the quencher and the fluorescent molecules, increased temperature results in reduced complex stability, and thus reduced quenching. In contrast, an increase in temperature results in increased molecular collisions between the quencher and the fluorescent molecules, and thus an increase in quenching [34].

**Figure 6:**
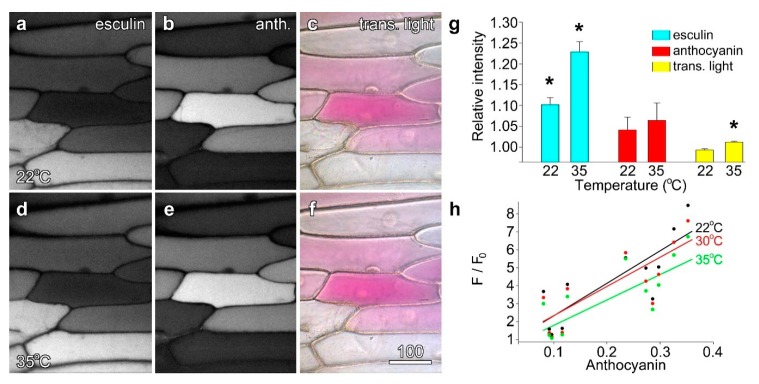
The quenching of esculin fluorescence was temperature dependent. Esculin (**a**,**d**) and anthocyanin fluorescence (**b**,**d**), along with false colour transmitted light (**c**,**f**), were collected at 22 °C (**a**–**c**) and at 35 °C. Esculin fluorescence was slightly but measurably higher at 35 °C (**d**). Bar in (**f**) = 50 µm for (**a**–**f**). (**g**) Quantification of esculin fluorescence (blue bars), and anthocyanin measured with fluorescence (red) and by transmitted light ratios (yellow). Data are ratios averaged for *n* = 12 individual cells, comparing values at the raised temperature to 22 °C. Asterisks indicate values significantly different from 1.00; Student’s *t*-test, *p* < 0.05. (**h**) Stern–Volmer plot (F/F_0_) for esculin in the presence of anthocyanin showing the temperature-dependence of quenching.

Onion epidermal cells loaded with the sugar analogue esculin were imaged at 25 °C, and then re-imaged using the same conditions at 30 °C and 35 °C. While anthocyanin fluorescence and cell colour remained unchanged, esculin fluorescence increased visibly although not dramatically (Figure 6a,d). When quantified, this temperature-dependent increase was confirmed: at 30 °C esculin fluorescence was ~10% higher than at 22 °C, and at 35 °C, ~22% higher, with both values being significant (Figure 6g). However, while anthocyanin fluorescence also increased marginally at higher temperatures (Figure 6b,e), these changes were not significant, and overall cell colour did not change between the two temperatures (Figure 6c,f). Further, as changes in anthocyanin fluorescence occurred solely in cells with low anthocyanin and high esculin fluorescence, they might indicate low levels of cross-talk between the fluorophores. Quantification of individual cells plotted against modified transmitted light ratios in Stern–Volmer plots showed that straight line best-fits decreased at higher temperatures confirming the presence of static quenching (Figure 6h).

## 3. Discussion

Anthocyanins are suggested to protect cells against reactive oxygen species (ROS) such as singlet oxygen, superoxide and hydrogen peroxide formed during photosynthesis and respiration [14] that can cause cellular damage to membranes, proteins and nucleic acids as they contain unpaired electrons [35]. Antioxidants delocalise these unpaired electrons and can, therefore, mitigate cellular damage [36]. The anthocyanin flavylium cation scavenges ROS both in vitro [15,16] and, it is suggested, in vivo [17]. For anthocyanins to be antioxidants, they need to interact directly with ROS. However, anthocyanins are synthesised in the cytoplasm, and then transported to the vacuole post-synthesis [12]. Because intracellular ROS are mainly produced by chloroplasts and mitochondria [18], the physiological significance of vacuolar anthocyanins as antioxidants has been debated [17,18]. However, some membrane permeable ROS including hydrogen peroxide may enter the vacuole where anthocyanin-based electron scavenging might occur [35], and dynamic tubular vacuoles extending through the cytoplasm may facilitate this [7].

The initial goal of this project was to determine whether anthocyanin might act as an antioxidant in vivo, preventing the photobleaching of fluorescent molecules in a manner similar to anti-fade agents such as *p*-phenylenediamine [22]. Thus, the experiments were designed to extend the work of Gould and colleagues who demonstrated in *Pseudowintera* that would-induced hydrogen peroxide, as measured with cytoplasmic loaded dichlorofluorescein, dissipated more rapidly in red, anthocyanic tissue than in non-coloured tissue [19]. The present study aimed to replicate these experiments using the more tractable onion inner epidermis where cell monolayers with patches of anthocyanic cells can be prepared with minimal wounding. However, experiments in which the anti-oxidant effects of anthocyanin on fluorescent dyes loaded into the vacuole were impractical because of fluorescence quenching. As vacuolar and cytoplasmic physiology appeared to be similar in anthocyanic and non-anthocyanic cells, it is assumed that direct quenching of the fluorescence of the exogenous dye by anthocyanin is happening. This is because vacuolar anthocyanin interfered with the fluorescence of the vacuole-targeted dyes loaded through the different pathways including acid-dependent loading (acridine orange) [27], probenecid-sensitive anion transporters (carboxyfluorescein, fluorescein and monochlorobimane) [23,24,25,26] and sugar transporters (esculin) [28]. With regards to the sugar transporter-dependent uptake of esculin, this has been described in the literature as being limited in monocots [28], although the initial analysis that showed this, only grass species were tested [37].

### 3.1. The Mechanism(s) Underlying Anthocyanin Quenching Phenomena

Quenching phenomena in which the fluorescence of a sample are decreased can be divided into static and dynamic quenching effects. Dynamic quenching involves collisions between the quenching molecule and the excited fluorophore which result in the non-radiative transfer of energy from the fluorophore to the quencher. In static quenching, however, a complex is formed between the quencher and the fluorophore that prevents the excitation of the fluorophore in the first place [34]. While the quenching activity of anthocyanin has not, seemingly, been demonstrated before in vivo, there are indications that this effect does occur in vitro. Anthocyanins at concentrations in the micromolar range will quench the intrinsic protein fluorescence from 280 nm excitation of tryptophan and tyrosine residues in β-lactoglobulin [38] and BSA [39], and in both cases the characteristics of the quenching, notably a reduction in quenching as temperature increased, were consistent with static quenching. Similar evidence for static quenching of intrinsic protein fluorescence was also observed for a range of polyphenol compounds [40]. As overall anthocyanin concentrations in red onions are in the order of 200 mg per kilogram fresh weight [21,41], which corresponds to an average concentration in the millimolar range across the entire onion bulb, the concentrations of anthocyanins present in the vacuole of the epidermal cells would be more than sufficient to induce similar quenching effects.

### 3.2. Future Directions

Several possible approaches exist that might be used to further investigate the effects of anthocyanin quenching. Single cell metabolomic approaches have been developed in which the vacuolar contents of individual cells can be assessed, with such approaches being highly applicable to the large epidermal cells of onions, detecting variations in anthocyanin content [42,43] and these might be used to confirm the effects of quenching. However, fluorescence lifetime imaging (FLIM) which is often used to study dynamic quenching which reduces the fluorescence lifetime of the quenched molecule [44] might not prove useful as fluorescent lifetimes are not typically reduced by static quenching [34]. FLIM has already been applied to anthocyanin, with Arabidopsis anthocyanin showing a pH-dependent lifetime of 0.1 to 0.5 nanoseconds [45], and it might be that a lack of a change in carboxyfluorescein lifetime in the presence of anthocyanin would confirm that static quenching. In vivo quenching of GFP by anthocyanin might also be investigated. For such experiments, the expression of vacuolar-targeted GFP through the commonly-used transient expression technology of the gene gun would not be suitable because of the widely varying expression levels achieved with the different transformation events. Instead, the non-trivial exercise of generating stable transformants of onion [46] or garlic [47] lines exhibiting epidermal anthocyanin, and expressing a vacuolar-targeted GFP might need to be used. However, stably transformed Arabidopsis lines expressing vacuolar targeted GFP might be induced to form anthocyanins through high sucrose treatments and other abiotic stresses [45,48,49].

## 4. Materials and Methods

### 4.1. Plant Material

Red onions were purchased from local shops. Red onion bulbs contain anthocyanin in the outer epidermis of all except the very inner layers, but the inner epidermis is mainly pigment-free except for occasional small patches of anthocyanin-containing cells. The outer, senescing layers of bulb tissue were discarded and experiments performed with epidermal peels (20 × 20 mm) of the inner epidermis of the inner leaves that contained patches of colouring. Samples of other plant species with variegated epidermal anthocyanin were collected from the University’s greenhouses.

### 4.2. Fluorescent Dyes

Dye stock solutions were prepared and stored frozen in dimethylsulfoxide (DMSO) (Table 1) and diluted in distilled water for incubations, except for esculin which was dissolved directly in water and used at a saturated concentration of 30 mM. Epidermal peels were floated mesophyll-side downwards on the dye solutions for up to 6 h, rinsed briefly by floating on distilled water, and then observed by confocal microscopy. The DMSO concentration of all solutions including controls was adjusted to 0.5% (*v/v*), except in the case of monochlorobimane where the hundred-fold dilution from the stock solution resulted in a final DMSO concentration of 1.0% (*v/v*).

### 4.3. Confocal Microscopy

Epidermal cells were imaged by confocal microscopy with either a 20 × NA 0.7 glycerol immersion lens (SP5 confocal, Leica, Wetzlar, Germany) or with a 30 × NA 1.05 silicon oil immersion lens (FV1000 confocal, Olympus, Tokyo, Japan). Four-fold line averaging and line-by-line sequential scanning for different excitation wavelengths, with sequential laser illumination was used to excite different fluorescent dyes (Table 1). Colour transmitted light images were prepared by combining transmitted light images collected with red, green and blue lasers, all in the absence of polarising filters [7,30]. As anthocyanin is only weakly fluorescent [7], anthocyanin imaging required high laser power with the green laser. Fluorescent dyes typically required only low laser intensities. Single optical sections were collected near the cortex of the cell as high anthocyanin concentrations could attenuate fluorescence towards the centre of the vacuole. Single vertical sections in the XZ plane were also collected to generate sections through the epidermis. For temperature controlled experiments, an on-stage incubator (Solent Scientific, Portsmouth, UK) was used to raise sample temperature above the room temperature of 22 °C.

Fluorescence emission spectra for carboxyfluorescein (lambda scanning) were recorded with 488 nm excitation and with emissions recorded from 500 to 700 nm with a 10 nm wide window and with a 4 nm step size. In vivo spectra were calculated for individual cells and normalised to 100, before being pooled according to transmitted light ratios (see below) and averaged.

### 4.4. Image Quantification

Three or more replicate experiments were run over multiple days for quantification experiments, but because there was variability in dye uptake and laser power between days, the data presented are limited to representative experiments conducted on single days. Fluorescence was quantified by selecting a region of interest in the vacuole, and calculating average image intensity in ImageJ at the same location for all the concurrently recorded images using the ‘*plot Z axis profile*’ function. To quantify dyes in the cytoplasm, small regions were selected for each cell and the values averaged. Background fluorescence was subtracted for all fluorescence images prior to processing. Transmitted light absorbance ratios were calculated as the amount of 633 nm (red) light transmitted by cells relative to the amount of 561 nm (green) light transmitted. This removed variations caused by shading or wrinkling within a sample, and removed variations in light levels between samples. Stern–Volmer plots, used to investigate quenching effects, used anthocyanin concentration as the dependent variable, with this defined either as the anthocyanin fluorescence or as a modified transmitted light ratio in which a constant value, the ratio for an anthocyanin-free cell, was subtracted from the ratio for each individual cell.

To investigate the quenching response, Stern–Volmer plots [34] were calculated for dyes in which a value, F/F_0_, was calculated for individual cells, with this value defined as being equal to the maximum dye fluorescence seen in any cell divided by the dye fluorescence. This was plotted against anthocyanin concentration, which was defined either as anthocyanin fluorescence, or as a modified transmitted light ratio which was defined as the transmitted light ratio for a cell minus the transmitted light ratio for the cell with the least anthocyanin. This later definition was preferred, as anthocyanin fluorescence intensity was non-linear at higher anthocyanin concentrations.

### 4.5. Image Processing

Images were processed in Adobe Photoshop version CS10 using contrast and gamma tools.

## 5. Conclusions

The vacuole of the inner epidermis of red onions sometimes become red, forming anthocyanin. As the presence of vacuolar anthocyanin causes a reduction in the fluorescence from a wide range of fluorescent dyes that can be loaded into the vacuole, and as there is no evidence for differences in metabolism between red and white cells that might explain differential loading, it is evident that the presence of anthocyanin causes quenching of the exogenous fluorescent dyes. Furthermore, as the anthocyanin-dependent quenching of fluorescence from the sugar analogue esculin decreases at higher temperatures, this effect if an example of static quenching. Similar effects have been demonstrated for anthocyanin in vitro. However, these data also suggest that some caution needs to be taken in the interpretation of experiments in which fluorescent dyes are loaded into plant cells containing anthocyanin.

## Figures and Tables

**Table 1:** Fluorescent dyes and proteins.

Fluorophore	Source	Stock ^1^ (mM)	Used (µM)	Loading (Hours)	Excitation ^2^ (nm)	Emission (nm)
*esterase released dyes*						
fluorescein diacetate	Invitrogen	10	10	6	488	500–530
carboxyfluorescein diacetate	Invitrogen	100	10	6	458, 488 **^3^**	500–530
BCECF-AM ester	Invitrogen	10	10	6	488	500–530
Cell Tracker green (5-chloro-methylfluorescein diacetate)	Invitrogen	10	10	6	488	500–530
*other enzyme-released dyes*						
monochlorobimane	Fluka	100	1000	6	405	420–470
Cell Tracker blue (CMAC)	Invitrogen	10	100	6	405	420–470
*weak acid dyes*						
acridine orange	Sigma	50	20	6	488	500–530
*sugar analogues*						
esculin	Sigma	30	--	4 h	405	420–460
*tonoplast membrane probes*						
MDY-64	Invitrogen	10	10	0.1	473	480–530
Lysotracker Red DND-99	Invitrogen	0.2	0.2	0.1	559	570–600
*Plant-generated molecules*						
anthocyanin	--	--	--	--	561	570–650 **^4^**

Note: **^1^** Stock solutions prepared in DMSO, except for esculin where the 30 mM solution in water was used on cells. **^2^** Excitation wavelengths of 405, 458, 488, and 561 nm are for the Leica SP5 confocal microscope whereas excitations of 405, 473, and 559 nm refer to the Olympus FV1000 confocal. **^3^** For ratiometric imaging of pH, dual excitation at 458 and 488 nm was used. **^4^** Anthocyanin imaged from 620 to 700 nm when co-imaged with the red fluorescent dye Lysotracker Red.

## Supplementary Materials

The following are available online at https://www.mdpi.com/2223-7747/8/12/597/s1; Figure S1: Vacuolar dye fluorescence is lower in the presence of anthocyanin., Figure S2: Vacuolar carboxyfluorescein fluorescence is lower in anthocyanic cells from other plant species., Figure S3: Vacuolar fluorescence is lower in the presence of anthocyanin.
