# Supplementary material for: Anthocyanin in the Vacuole of Red Onion Epidermal Cells Quenches Other Fluorescent Molecules
Source: Plants (Basel). 2019 Dec 12;8(12):596. doi: 10.3390/plants8120596 (PMC6963288; doi:10.3390/plants8120596)
Supplement: Supplementary file 1 [file plants-08-00596-s001.zip › plants-661942-proofed/Collings - Supplementary Figures.docx]

**
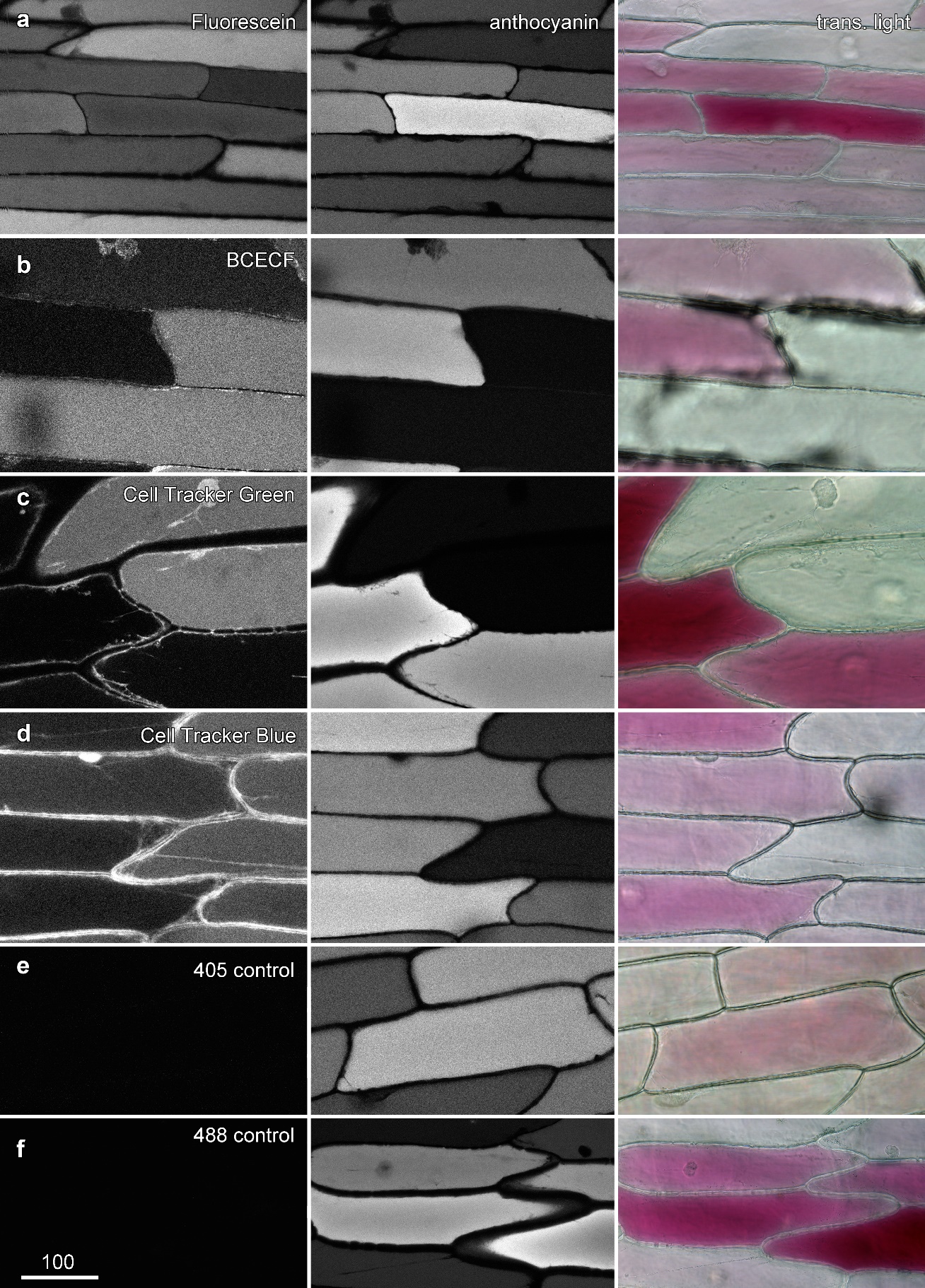
**

**Figure S1.** Vacuolar dye fluorescence is lower in the presence of anthocyanin. Epidermal peels loaded with dyes (6 h) were imaged with sequential scanning. Images are confocal optical sections of dye fluorescence (left column) and anthocyanin fluorescence (central column), and a colour transmitted light image (right column). **a** FDA imaged with blue excitation. **b** BCECF imaged with blue excitation. **c** 5-chloromethylfluorescein (Cell Tracker Green) imaged with blue excitation. **d** Cell Tracker blue imaged with violet excitation. **e**, **f** Control cells (unstained) imaged with 405 nm violet (**H**) and 488 nm blue (**I**) excitation showed no fluorescence in the dye channels when imaged using similar conditions. Bar in **f** = 100 µm for all images.


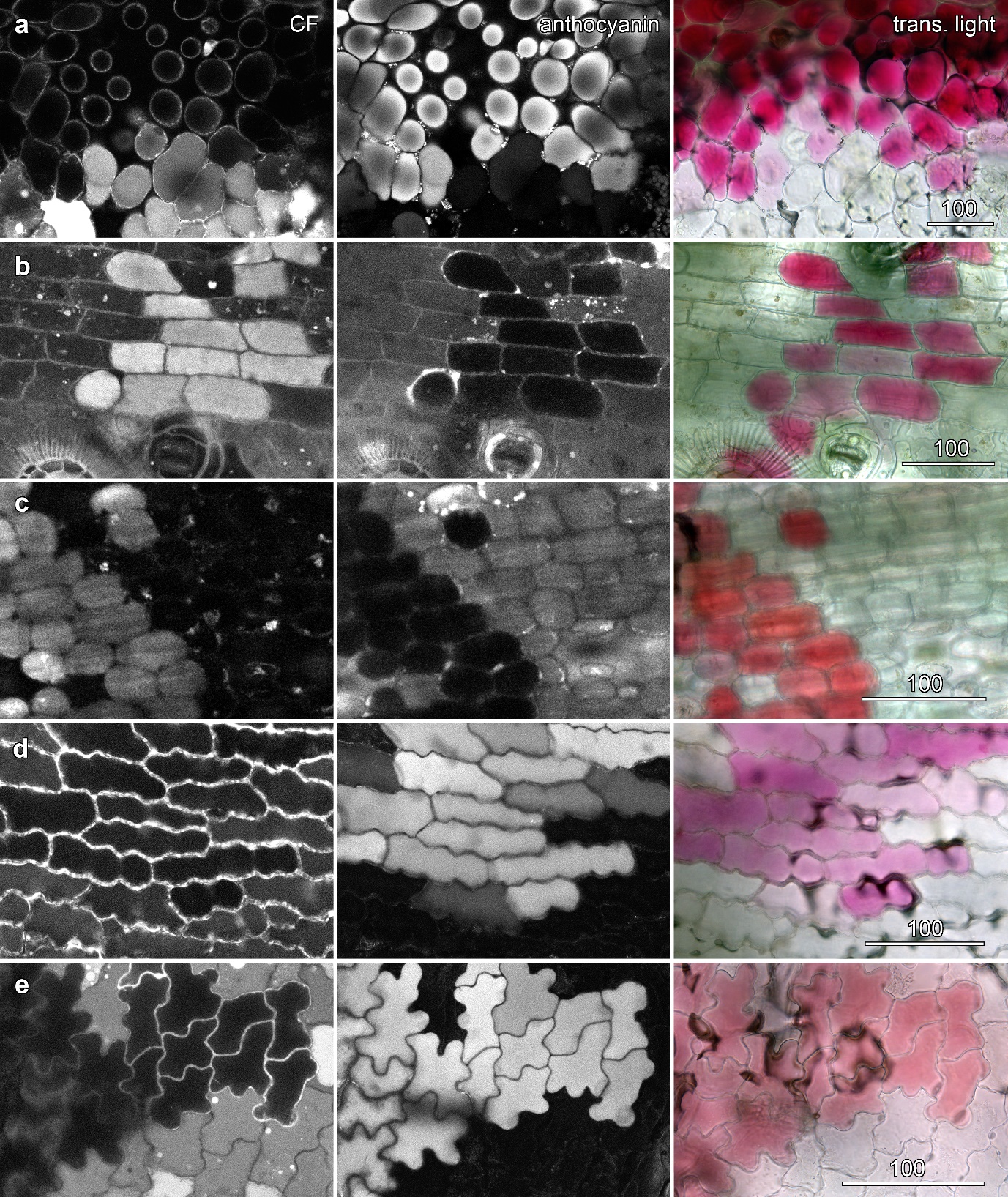


**Figure S2.** Vacuolar carboxyfluorescein fluorescence is lower in anthocyanic cells from other plant species. Epidermal peels were loaded with carboxyfluorescein diacetate (6 h) and imaged with sequential scanning. Images are confocal optical sections for carboxyfluorescein (left column), and anthocyanin fluorescence (central column), along with colour transmitted light images (right column). **a** Lower leaf epidermis of a variegated coleus (*Coleus* sp.). **b** Mesophyll cells from a partial epidermal peel from the leaves of a bromeliad (*Neoregelia* sp). **c** Mesophyll cells from a partial epidermal peel from the leaves of a *Dracaena* sp. **d** Lower epidermis of the petals of a cyclamen (*Cyclamen* sp.). **e** Lower epidermis of flower petals from a columbine (*Aquilegia* sp.). Bars equal 100 µm.


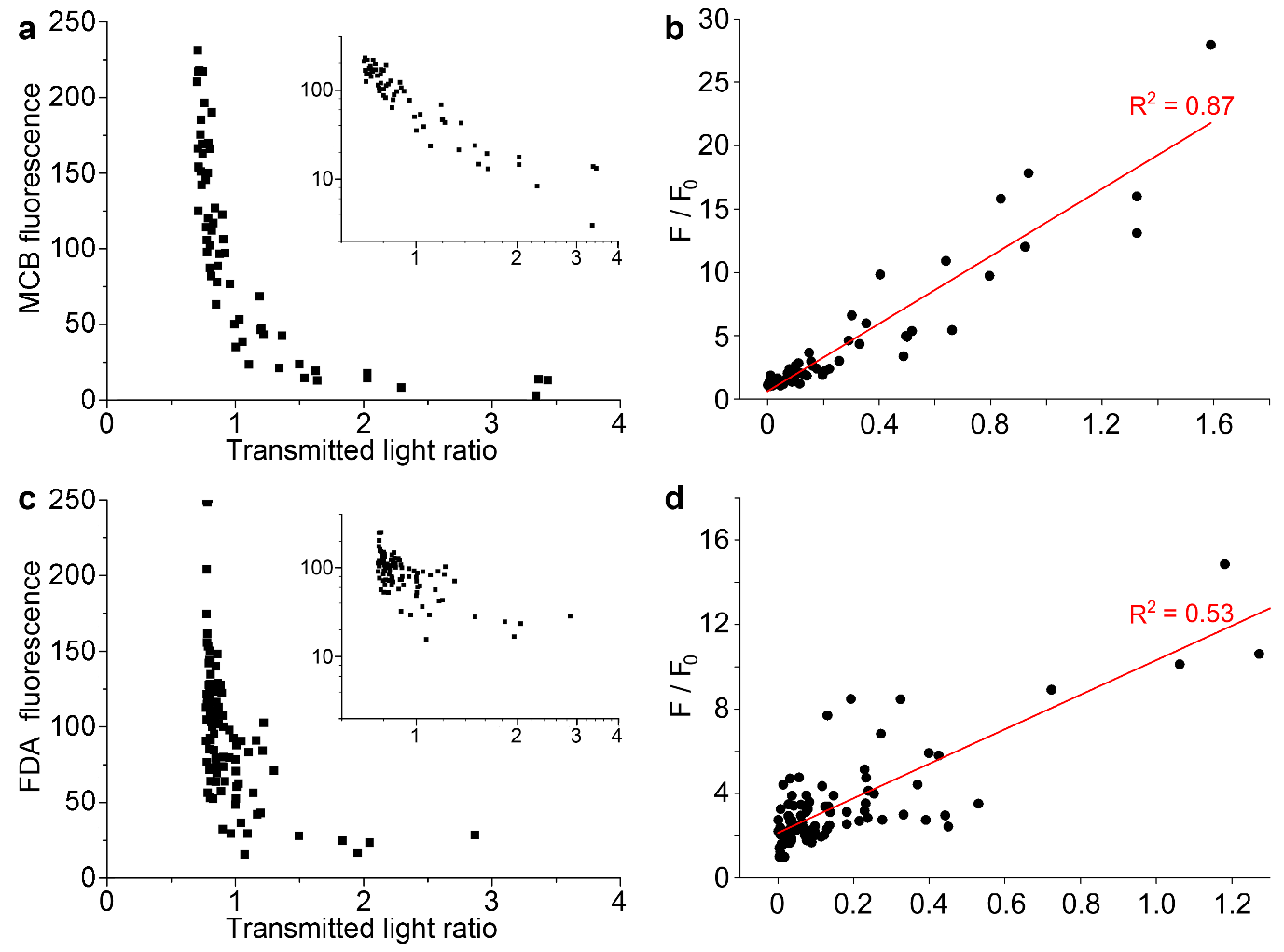


**Figure S3.** Vacuolar fluorescence is lower in the presence of anthocyanin. Individual cells are shown from single representative experiments that had been treated with either monochlorobimane (MCB) (**a**, **b**) or fluorescein diacetate (FDA) (**c**, **d**) for 6 h. **a** Vacuolar monochlorobimane fluorescence showed a strong negative correlation with the transmitted light ratio. The inset shows the same data set re-plotted on a double-log plot. **b** Stern-Volmer plot (F / F_0_) for monochlorobimane in the presence of anthocyanin. **c** Vacuolar fluorescein fluorescence showed a strong negative correlation with the transmitted light ratio. The inset shows the same data set re-plotted on a double-log plot. **d** Stern-Volmer plot (F / F_0_) for fluorescein in the presence of anthocyanin.
